# Unusual Left Periclavicular Cutaneous Lymphatic Fistula After Port Explantation Without Lymph Vessel Injury: Imaging and Interventional Treatment

**DOI:** 10.1007/s00270-021-02840-2

**Published:** 2021-04-29

**Authors:** Julia Wagenpfeil, Ulrike Attenberger, Claus Christian Pieper

**Affiliations:** Department of Diagnostic and Interventional Radiology, University Hospital Bonn, Venusberg-Campus 1, 53127 Bonn, Germany

**Keywords:** Lymphatic fistula, Magnetic resonance lymphangiography, Embolization

## Abstract

Complex oncological treatment can be associated with lymphatic vascular injury that is burdened by considerable morbidity. Lymphatic imaging and interventional techniques offer new minimally invasive treatment options. We report the case of a 59-year-old woman with an unusual lympho-veno-cutaneous fistula, diagnosed by magnetic resonance lymphangiography and treated by minimally invasive embolization therapy and venous recanalization.

## Introduction

Lymphatic fistulas are rare, difficult to treat and potentially fatal complications of oncologic surgery or central venous catheterization. Lymphatic fistulas after surgery usually occur due to direct lymph vessel injury (e.g. of the thoracic duct) [1]. In cases with continued lymphatic discharge, a conservative treatment attempt (e.g. medium chain triglyceride diet, parenteral nutrition) is indicated. However, intractable fistulas may also require surgical or—nowadays—interventional treatment [2].

New techniques to visualize lymph vessels and perform targeted minimally invasive treatment have been established [2]. Especially MR-lymphangiography (MRL) has proven to be a useful tool that can visualize the central lymphatic system (CLS) and can—when performed as dynamic MRL—also provide functional flow information [3–5].

We report a case of a 59-year-old woman with cutaneous high-volume chylolymphatic discharge due to an unusual lympho-veno-cutaneous fistula without any direct lymph vessel injury, demonstrating the importance of selective lymphatic imaging to enable targeted therapy.

## Case Report

A 59-year-old woman with advanced high-grade ovarian cancer presented with a high-volume chylolymphatic cutaneous fistula with a drainage volume of 1000–2000 ml chylous fluid (triglyceride levels > 500 mg/dl) per day from a left periclavicular wound after explantation of a pectoral port catheter system. Port explantation became necessary due to acute venous thrombosis of the left subclavian, internal jugular and brachiocephalic vein with oedema of the left arm.

Initially conservative treatment was tried for 2 weeks with anticoagulation with heparin and parenteral nutrition at an outside hospital. However, drainage volume was unaffected and conservative therapy was ultimately unsuccessful with the patient developing progressing cachexia. Clinically the cause of lymphatic leakage remained unclear, as a post-operative lymph vessel injury was unlikely due to only superficial incision for port extraction. The patient was referred to our hospital for further treatment. We performed interstitial transpedal magnetic resonance lymphangiography (MRL). Transpedal MRL is described in detail elsewhere [4]. In short, a diluted gadolinium-based contrast agent (Gadovist, Bayer Healthcare) was injected intradermally into the interdigital web-spaces of both feet. After active leg movement by the patient, high-resolution T1-weighted images were acquired in a coronal plane. MR-lymphangiograms showed normal enhancement of the CLS with an intact thoracic duct running from the cisterna chyli to the left venous angel. There was no visible direct lymph vessel injury.

**Fig. 1:**
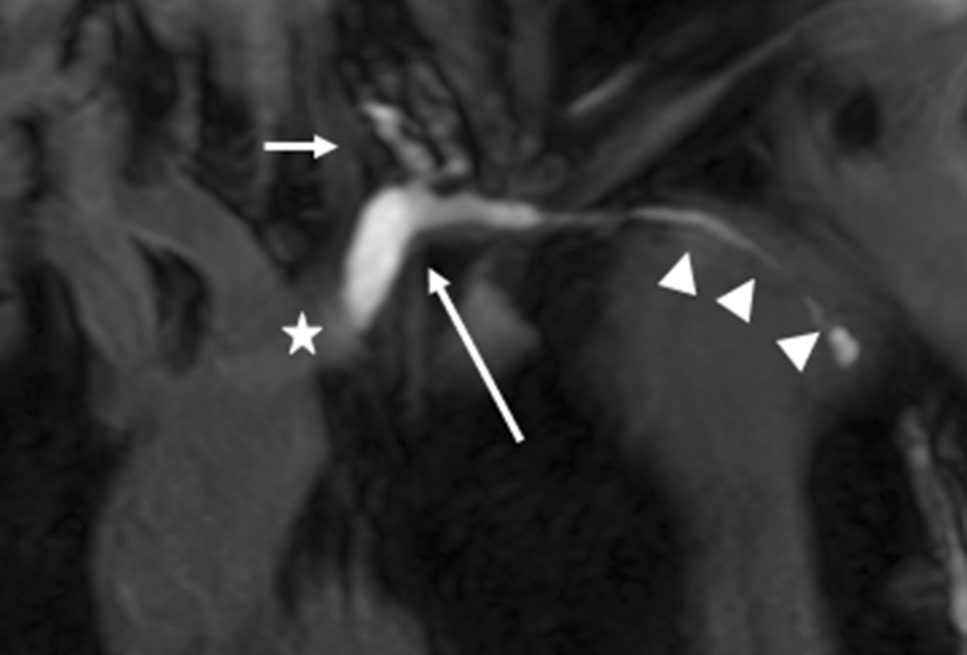
Coronal, T1-weighted MR-lymphangiogram demonstrating enhancement of the terminal thoracic duct (short arrow) draining into the left subclavian vein (long arrow) isolated by proximal and distal thrombotic occlusion (central occlusion: asterisk). From this isolated venous segment the contrast agent drains through the former port catheter tract (arrow heads) to the skin

The venous segment into which the thoracic duct drained was recanalized, but isolated from central and distal venous blood flow by persistent thrombosis of the brachiocephalic and axillary vein. The port catheter was previously inserted into this segment, resulting in lymph flow from the isolated venous segment through the established catheter tract into the port recess with subsequent cutaneous leakage (Fig. 1). The cause of the cutaneous chylolymphatic discharge was therefore identified as a lympho-veno-cutaneous fistula.

**Fig. 2:**
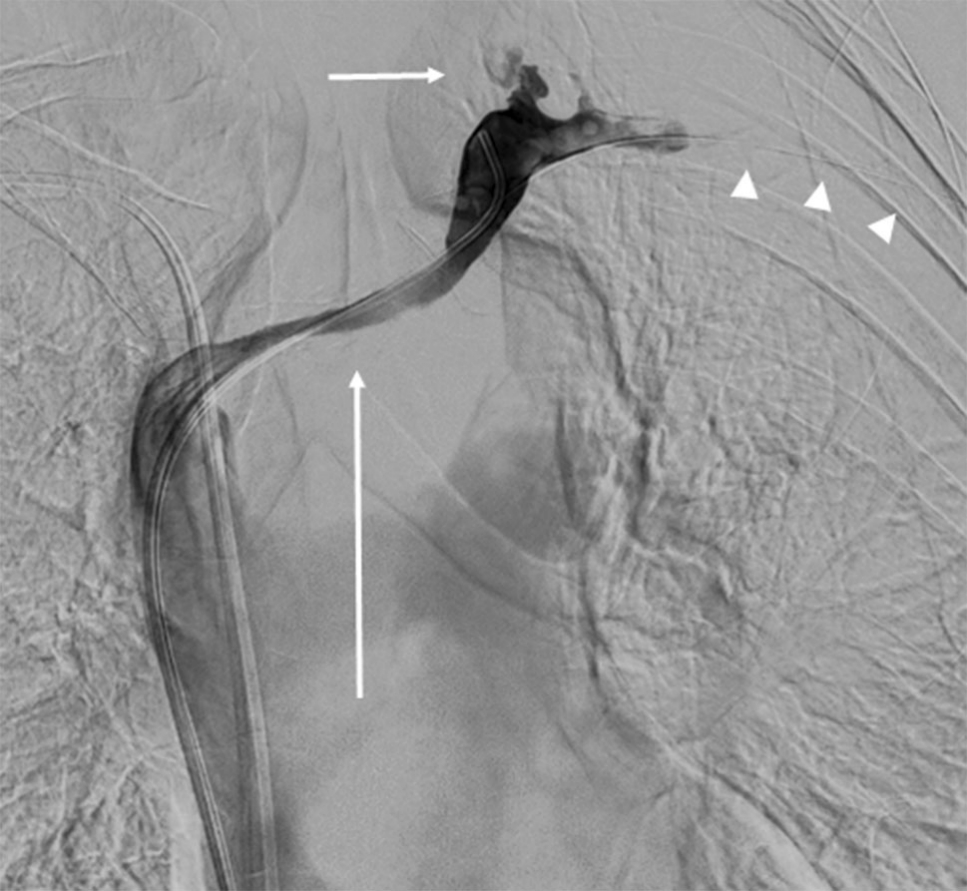
Corresponding intra-interventional digital-subtraction angiography during recanalization of the left brachiocephalic vein (long arrow). There is reflux of contrast-agent into the terminal thoracic duct (short arrow). Note also the indwelling microcatheter in the fistula tract (i.e. former port catheter tract) (arrowheads)

Interventional treatment was planned with the intention of keeping central lymphatic drainage intact. Through a right femoral venous access, a 5F diagnostic catheter was used to pass the venous occlusion of the brachiocephalic vein. Additionally, the fistula was cannulated from the cutaneous porus using a microcatheter (Renegade, Boston Scientific) and microwire (Transend, Boston Scientific). Digital-subtraction angiography confirmed MR-lymphangiographic findings. Central venous drainage was recanalized from by percutaneous transluminal angioplasty (Fig. 2) and implantation of a 12 × 60 mm bare metal stent (Zilver, Cook Medical). The fistula was embolized with microcoils (Hilal and Tornado, Cook Medical) and tissue adhesive (1:1 mixture of Histoacryl [Braun] and Lipiodol [Guerbet]).

Lymphatic leakage ceased completely immediately after the intervention. There was no recurrence of leakage or post-interventional sequela during a follow-up of 4 weeks. After this period, the patient succumbed to her advanced stage ovarian cancer.

## Discussion

This unusual case of an intractable lympho-veno-cutaneous fistula, initially thought to be due to post-surgical lymph vessel injury with high-volume chylolymphatic discharge, demonstrates the value of novel lymphatic imaging and interventional techniques. Although the incidence of post-surgical chylolymphatic leakage after head and neck surgery is low, the consequences of this complication such as malnutrition, infection and immunosuppression can be severe and life threatening [1]. It is well known that in at least 30% of patients there are considerable anatomical variations of the CLS [6] that not only predispose for intraoperative lymph vessel injury, but also complicate management. Establishing the cause of leakage therefore is imperative for adequate treatment. Techniques for visualization of lymphatic vessels have evolved rapidly in recent years. Conventional X-ray lymphangiography has been the mainstay of lymphatic imaging for decades, but can be time-consuming and associated with complications. MR-lymphangiography is a novel, non-invasive alternative method to depict the CLS. In the presented case, MRL demonstrated that lymphatic leakage in fact did not result from lymph vessel injury. The thoracic duct drained the lymph from the lower body into an isolated venous segment with the only patent out-flow being an established tract of a former indwelling port catheter to the skin.

Conservative management is usually the first-line therapy of post-operative lymphatic fistulas [2]. In this specific case, drawbacks of conservative management include long therapy periods and long hospitalization, risk of skin maceration and infection. In oncologic patients necessary chemotherapy often has to be paused while chylolymphatic leakage persists. Traditionally surgical treatment (e.g. thoracic duct ligation) is indicated after failure of conservative treatment [1, 7]. However, surgical treatment of lymphatic leakage can be associated with considerable morbidity and mortality (up to 38% and 25%, respectively) [2] and would not have been feasible in our patient with poor general condition. Nowadays interventional techniques are available (e.g. lymph vessel embolization) with lower rates of mortality and morbidity [2]. In the presented case occlusion of central lymphatics—as would traditionally have been performed—was not necessary to treat the cutaneous leakage due to the lack of an actual lymph vessel injury. By precisely depicting the pathology as being a lympho-veno-cutaneous fistula caused by venous obstruction, MRL enabled targeted, minimally invasive therapy by recanalization of central venous run-off and selective occlusion of the fistula tract. Central lymphatic drainage remained patent, so that possible associated complications of central lymphatic obstruction (e.g. lymphedema) could be avoided.

In conclusion, management of post-surgical chylolymphatic leakage remains challenging. Dedicated lymphatic imaging in combination with a growing range of interventional treatment options enable individualized and targeted treatment of these lymphatic pathologies.

## References

[1] Campisi CC, Boccardo F, Piazza C, Campisi C (2013). Evolution of chylous fistula management after neck dissection. Curr Opin Otolaryngol Head Neck Surg.

[2] Pieper CC, Hur S, Sommer CM, Nadolski G, Maleux G, Kim J, Itkin M (2019). Back to the future: Lipiodol in lymphography-from diagnostics to theranostics. Invest Radiol.

[3] Krishnamurthy R, Hernandez A, Kavuk S (2015). Imaging the central conducting lymphatics: initial experience with dynamic MR lymphangiography. Radiology.

[4] Pieper CC, Feist A, Schild HH (2020). Contrast-enhanced interstitial transpedal MR Lymphangiograpy for Thoracic Chylous Effusions. Radiology.

[5] Itkin M, Nadolski GJ (2018). Modern techniques of lymphangiography and interventions: Current status and future development. Cardiovasc Interven Radiol.

[6] Phang K, Bowman M, Phillips A, Windsor J (2014). Review of thoracic duct anatomical variations and clinical implications. Clin Anat.

[7] Nair SK, Petko M, Hayward MP (2007). Aetiology and management of chylothorax in adults. Eur J Cardiothorac Surg.

